# Neutrophil Extracellular Traps Induce Organ Damage during Experimental and Clinical Sepsis

**DOI:** 10.1371/journal.pone.0148142

**Published:** 2016-02-05

**Authors:** Paula Giselle Czaikoski, José Maurício Segundo Correia Mota, Daniele Carvalho Nascimento, Fabiane Sônego, Fernanda Vargas e Silva Castanheira, Paulo Henrique Melo, Gabriela Trentin Scortegagna, Rangel Leal Silva, Romualdo Barroso-Sousa, Fabrício Oliveira Souto, Antonio Pazin-Filho, Florencio Figueiredo, José Carlos Alves-Filho, Fernando Queiróz Cunha

**Affiliations:** 1 Department of Pharmacology, Ribeirão Preto Medical School, University of Sao Paulo, Ribeirao Preto, Brazil; 2 Department of Clinics, Ribeirão Preto Medical School, University of Sao Paulo, Ribeirao Preto, Brazil; 3 Department of Biochemistry and Immunology, Ribeirão Preto Medical School, University of Sao Paulo, Ribeirao Preto, Brazil; 4 Department of Pathology, School of Medicine, University of Brasilia, Brasilia, Brazil; University of Florida, UNITED STATES

## Abstract

Organ dysfunction is a major concern in sepsis pathophysiology and contributes to its high mortality rate. Neutrophil extracellular traps (NETs) have been implicated in endothelial damage and take part in the pathogenesis of organ dysfunction in several conditions. NETs also have an important role in counteracting invading microorganisms during infection. The aim of this study was to evaluate systemic NETs formation, their participation in host bacterial clearance and their contribution to organ dysfunction in sepsis. C57Bl/6 mice were subjected to endotoxic shock or a polymicrobial sepsis model induced by cecal ligation and puncture (CLP). The involvement of cf-DNA/NETs in the physiopathology of sepsis was evaluated through NETs degradation by rhDNase. This treatment was also associated with a broad-spectrum antibiotic treatment (ertapenem) in mice after CLP. CLP or endotoxin administration induced a significant increase in the serum concentrations of NETs. The increase in CLP-induced NETs was sustained over a period of 3 to 24 h after surgery in mice and was not inhibited by the antibiotic treatment. Systemic rhDNase treatment reduced serum NETs and increased the bacterial load in non-antibiotic-treated septic mice. rhDNase plus antibiotics attenuated sepsis-induced organ damage and improved the survival rate. The correlation between the presence of NETs in peripheral blood and organ dysfunction was evaluated in 31 septic patients. Higher cf-DNA concentrations were detected in septic patients in comparison with healthy controls, and levels were correlated with sepsis severity and organ dysfunction. In conclusion, cf-DNA/NETs are formed during sepsis and are associated with sepsis severity. In the experimental setting, the degradation of NETs by rhDNase attenuates organ damage only when combined with antibiotics, confirming that NETs take part in sepsis pathogenesis. Altogether, our results suggest that NETs are important for host bacterial control and are relevant actors in the pathogenesis of sepsis.

## Introduction

Sepsis is a systemic inflammatory response syndrome caused by the inability of the host to locally restrict an infection [1]. Sepsis is one of the most common causes of death in healthcare units worldwide, and the incidence of sepsis is increasing. Our poor understanding of the pathophysiology of sepsis explains this critical situation [2].

The successful clearance of pathogens to avoid their spreading into the circulation depends on efficient neutrophil recruitment to the infection site and microbicidal activity of the emigrated cells. Neutrophil migration to the infection site is mainly mediated by G protein-coupled receptor signaling, including the CXCR subfamily, the leukotriene receptor and the C5a receptor [3, 4]. Moreover, the microbicidal activity of the emigrated neutrophils is mediated by oxygen- and/or nitrogen-derived free radicals and by microbicidal enzymes [5].

A frequent complication of sepsis is the development of organ dysfunction, including that of the heart, kidney and liver [6]. The pathogenesis of organ dysfunction during sepsis remains incompletely understood [7]. However, there is growing evidence that inflammatory mediators act synergistically with microorganism end products to induce systemic neutrophil activation, which contributes to their accumulation in distant organs and precludes their proper migration to the focus of infection. The activation of neutrophils causes their accumulation in the microcirculation, inducing local cytotoxicity mediated by oxygen and/or nitrogen-derived free radicals and cytotoxic enzymes, such as myeloperoxidase (MPO) and proteases [8,9].

During their activation, neutrophils also release a network of chromatin fibers associated with granule antimicrobial peptides and enzymes, such as MPO, elastase, and cathepsin G, known as neutrophil extracellular traps (NETs) [10]. NETs represent an innate defense mechanism; they immobilize and kill invading microorganisms to prevent their spreading and ensure a high local concentration of antimicrobial agents to degrade virulence factors and kill pathogens [10,11,12]. In addition to their host-protective role during infection [13,14,15], the excessive formation of NETs has also been observed in many pathological conditions, such as appendicitis [10], cystic fibrosis [16], thrombosis [17], acute lung injury [18,19], systemic lupus erythematosus [20,21] and small vessel vasculitis [22], diabetes [23] and sepsis [24, 25]. However, the role of NETs in the physiopathology of sepsis is not well understood [26, 27].

In the present study, we showed that NETs were systemically formed in sepsis and play a dual opposite role: on the one hand, NETs control the infection, but on the other hand, they induce tissue and organ damage. Accordingly, the degradation of NETs by recombinant DNase (rhDNase) increased the bacterial burden, but when combined with antibiotics to control the infection, this strategy attenuated the systemic inflammatory response, reduced endothelial and organ damage, and increased the survival rate of severely septic mice. Additionally, we present the first report that serum concentrations of cf-DNA, a surrogate marker of NETs levels, were positively correlated with sepsis severity and organ failure in human sepsis.

## Methods

### Patients

Peripheral blood samples were collected from 31 septic patients who were prospectively enrolled in the study within the first twenty-four hours of admission in the Emergency Department of a high-complexity hospital. All patients presented clinical or laboratory variables that fulfilled the criteria for sepsis [28]. Written informed consent was obtained from eight healthy volunteers included as controls and from each patient. The study was approved by the Human Subjects Institutional Committee of the Ribeirão Preto Medical School, University of Sao Paulo, under protocol number 5965/2009. Sepsis severity was evaluated by the Sequential Organ Failure Assessment (SOFA) score, as previously described [29,30]. Acute kidney injury (AKI) was analyzed following the criteria established by the Acute Kidney Injury Network (AKIN) [31], and Acute Respiratory Distress Syndrome (ARDS) was diagnosed and graded following the Berlin criteria [32].

### Mice

C57BL/6 mice were obtained from the Ribeirão Preto Medical School. All experiments were conducted in compliance with the Guide for the Care and Use of Laboratory Animals of the National Institutes of Health. The protocol was previously approved by the Animal Ethics Committee of the Ribeirão Preto Medical School, University of Sao Paulo, Sao Paulo, Brazil (Protocol number: 047/2010).

### Drugs

The following drugs were used: ertapenem sodium (Merck Research Laboratory, Whitehouse Station, NJ, USA); recombinant human DNase (rhDNase–Pulmozyme®, Roche–Genentech Inc., South San Francisco, California, USA); and Escherichia coli lipopolysaccharide (LPS, Sigma-Aldrich, St. Louis, MO, USA). Ertapenem sodium and LPS were dissolved in phosphate-buffered saline (PBS).

### CLP sepsis animal model and endotoxemia model

Sepsis was induced by cecal ligation and puncture (CLP) as described elsewhere [33]. Briefly, the mice were anesthetized intraperitoneally. (ip., 100 mg/kg ketamine; 10 mg/kg xylazine), and an incision was made in the abdomen. The cecum was exposed and ligated below the ileocecal junction, and a single puncture with an 18-g needle was made through the cecum. Control mice (sham) were submitted to the same procedures without cecal puncture. All animals received 1 mL of saline sc. immediately following surgery. In the endotoxemia model, mice received LPS (15 mg/kg, intravenously—iv.). The endotoxemia model allowed us to study multiple organ injury or dysfunction during the systemic inflammatory response in the absence of bacterial infection.

### Experimental protocols

For all survival experiments, the survival rate was determined every 12 h for 7 days. At this time, mice that showed signs of imminent death (i.e., inability to maintain upright position/ataxia/tremor and/or agonal breathing) were euthanized by ketamine/xylazine (>100/10 mg/kg, subcutaneously—sc., União Quimica, BR) overdose. At the end of 7 days, live mice were euthanized. The number of animals for survival curves was 5–10 per each group. For sample harvesting, mice were euthanized in a CO2 chamber.

Protocol 1—For the characterization of organ damage during sepsis, we used two experimental groups: S\sham and CLP-SS (mice with severe sepsis induced by CLP). Blood levels of the organ damage biomarkers creatine kinase MB isoenzyme (CK-MB), blood urea nitrogen (BUN) and aspartate aminotransferase (AST), tumor necrosis factor (TNF), cell-free DNA (cf-DNA), neutrophil extracellular traps (cf-DNA/MPO) and bacteria were determined at 3, 6, 12, or 24 h after sepsis induction. Neutrophil infiltration in lung tissue was estimated by MPO quantification at 3, 6 or 12 h after sepsis induction. There were 5 animals in each group and the experiment was repeated 2 times.

Protocol 2 –For the experiment with rhDNase treatment, we used three experimental groups: sham, CLP + Sal (severe sepsis treated with saline sc.) and CLP + rhDNase (severe sepsis treated with rhDNase sc.) The mice were treated with saline or rhDNase (10 mg/kg, sc.) pre- (10 min) and post- (4 h) sepsis induction. Six hours after sepsis induction, we evaluated the blood levels of organ damage biomarkers (CK-MB, BUN, and AST), cf-DNA/NET, and bacteria. In survival experiments, mice were treated as previously described and every eight hours after that. There were five animals per group and the experiment was repeated 2 times.

Protocol 3 –For the experiment with rhDNase treatment plus antibiotic therapy, we used three experimental groups: sham, CLP + Ant (severe sepsis treated with antibiotic plus saline) and CLP + Ant + rhDNase (severe sepsis treated with antibiotic plus rhDNase). Mice were treated with saline or rhDNase (10 mg/kg, sc. - 1 h and 8 h after the surgery) plus ertapenem antibiotic (30 mg/kg, sc. - 1 h after the surgery). Twelve hours after sepsis induction, we evaluated the blood levels of organ damage biomarkers (CK-MB, BUN, AST and endocan), cf-DNA/NET, TNF, IL-6, and MPO in lung tissue, and bacteria levels in the blood. We also quantified the bacterial load and the numbers of mononuclear leukocytes and neutrophils in the peritoneal lavage, as described elsewhere [34]. For the survival experiments, the mice were treated with saline or rhDNase (10 mg/kg, sc. - 1 h after the surgery and then every 8 h) plus ertapenem antibiotic (30 mg/kg, sc. - 1 h after the surgery and then every 12 h thereafter) up to day 3. There were 5 animals in each group and the experiment was repeated 2 times.

Protocol 4 –For the experiment with endotoxemic mice treated with rhDNase, we used three experimental groups: CTRL (naive), LPS + Sal (mice with endotoxic shock treated with saline sc.) and LPS + rhDNase (mice with endotoxic shock treated with rhDNase sc.). The mice were treated with saline or rhDNase (10 mg/kg, sc.) pre- (10 min) and post- (8 h) endotoxic shock. Twelve hours after endotoxic shock induction, we evaluated the blood levels of organ damage biomarkers (CK-MB, BUN, and AST), cf-DNA, TNF-α and MPO in lung tissue. We also evaluated the NETs deposition in kidney tissues 12 h after endotoxic shock induction. For the survival experiments, the mice were treated with saline or rhDNase (10 mg/kg, sc.) pre- (10 min) and post- (8 h) endotoxic shock and then every 8 h thereafter up to day 3. There were 5 animals in each group and the experiment was repeated 2 times.

Protocol 5 –For the experiment to determine whether rhDNase degrades cf-DNA in vitro in the serum of septic patients, 40 μL of serum was incubated with rhDNase (concentration: 100 μg of DNase/1 mL of serum) for 30 minutes at 37°C. This concentration was determined based on the corresponding concentration of DNase that was experimentally tested *in vivo*, which is equivalent to 100 μg/mL. Cell free-DNA was quantified in the serum using the Quant-iT™ PicoGreen® kit (Invitrogen) according to the manufacturer’s instructions, as previously described [35]. There were 5 animals per group and the experiment was repeated 2 times.

### Bacterial counts

The bacterial counts were determined as previously described [34]. Briefly, peritoneal lavage fluid and blood samples were harvested, plated on Muller-Hinton agar dishes (Difco Laboratories, Detroit, Michigan), and incubated for 24 h at 37°C.

### Cytokine determination

Cytokine and chemokine concentrations were measured by ELISA, as previously described [34]. The detection limit of the test for IL-6 was 60 pg/mL and for TNF was 1.95 pg/mL; samples with concentrations below these values were indicated as ND (not detected).

### cf-DNA quantification

The animals were anesthetized and whole blood was collected by cardiac puncture. The samples were centrifuged (15 min, 4°C, 500 x g), and the serum or plasma was collected. Cell free-DNA (cf-DNA) was quantified in the serum using the Quant-iT™ PicoGreen® kit (Invitrogen) according to the manufacturer’s instructions, as previously described [35]. The fluorescence (emission at 485-nm wavelength) intensity, a measure of the amount of DNA, was quantified by a fluorescence reader (FlexStation 3 Microplate Reader, Molecular Devices, California, USA). Cf-DNA concentrations were calculated, using calf thymus DNA (Sigma, Taufkirchen, Germany) as a standard.

### cfDNA/MPO (NET) quantification

The concentration of cf-DNA bound to MPO (cf-DNA/MPO), a constituent of NET, was also measured in the serum or plasma of septic mice. This method allowed the identification of NETs in the circulation of the animals. Briefly, an antibody bound to the 96-well flat-bottom plate captured the enzyme MPO, a constituent of NET, and the amount of DNA bound to the enzyme was quantified using the Quant-iT™ PicoGreen® kit (Invitrogen), as above.

### Blood biomarkers of organ injury analysis

The animals were anesthetized and whole blood was collected by cardiac puncture. The samples were centrifuged (15 min, 4°C, 500 g) and the serum was collected. The following biochemical parameters were measured in the serum as markers of multiple organ injury or dysfunction: liver injury was assessed by measuring the increase in serum concentrations of AST; renal dysfunction was evaluated by measuring the increase in BUN; and cardiac lesions were assessed by measuring CK-MB levels. The determinations were made using a commercial kit (Labtest, Lagoa Santa, Brazil). Serum endocan concentrations were measured by ELISA (Mouse Endocan DuoSet, R&D Systems). The detection limit of the test for endocan was 1.46 pg/mL.

### Lung tissue myeloperoxidase activity

Briefly, the animals were euthanized in a CO2 chamber after sepsis or endotoxemia induction. The myeloperoxidase activity in lung homogenates was assayed as previously described [34].

### Immunofluorescence

Samples of kidney tissue were collected from mice, embedded in Tissue-Tek® O.C.T.™, immediately frozen in 2-methylbutane (isopentane) resting in liquid nitrogen and stored at −70°C. Sections were cut to a thickness of 10–15 μm, fixed with 4% paraformaldehyde and stained with Hoechst 33342 (1 μg/mL, Molecular Probes), anti-MPO (1:50; sc-16128-R, Santa Cruz Biotechnology) and anti-DNA/histone H1 (1:200, MAB3864, Merck Millipore) antibodies, followed by anti-rabbit Alexa Fluor 594 (1:100; Molecular Probes) or anti-mouse Alexa Fluor 488 (1:400; Molecular Probes). Confocal images were taken with a Leica TCS SP5-AOBS microscope (Leica Microsystems, Mannheim, Germany); epifluorescence images were taken with a Zeiss Axioplan. Permeabilizing agents were not used, resulting in exclusive extracellular labeling. Assessment was made in a purely qualitative fashion to detect the presence or absence of NETs in the analyzed samples.

### Histological examination

The animals were euthanized 12 h after endotoxic shock induction, and the lungs, hearts, and livers were isolated and fixed by immersion in 10% paraformaldehyde, dehydrated and embedded in paraffin wax. Then, 5-μm-thick sections were stained with hematoxylin and eosin for histological examination. A semi-quantitative analysis was carried out in a blinded fashion by an experienced pathologist who was unaware of the groups (Figueiredo, F) to quantify the results, defined as: 0 = normal, + = mild, ++ = moderate, +++ = severe histological changes. The tissues and parameters assessed included the heart (edematous dissociation, congestion of the capillary, mononuclear and polymorphonuclear leukocytes attached to the vascular endothelium); the lungs (thickening of the septum, edema, congestion and intestinal leukocyte infiltration); and the liver (enlarged sinusoids, increased volume of endothelial cells, luminal leukocyte infiltration, Kupffer cell hypertrophy and hyperplasia).

### Statistical analysis

All of the data are reported as the means ± SEM of the values obtained from two independent experiments (n = 5 mice for each group, except for the survival experiments, in which 15 animals were in each group). The data were compared by analysis of variance (ANOVA) followed by the Bonferroni correction. Tukey’s test or Student’s t-test were used for unpaired values. Bacterial counts were analyzed by the Mann-Whitney U test. The survival rate was expressed as the percentage of live animals, and the Mantel-Cox log-rank test was used to compare the survival curves. Clinical data were summarized using the means ± SD or medians with interquartile ranges (IQRs). If variables were skewed, nonparametric tests were employed. Continuous variables were compared using t-tests, nonparametric tests for trends across ordered groups, the Mann-Whitney U-test, and the Kruskal-Wallis test. Spearman’s rank correlation coefficient (σ) was calculated to describe correlations between cf-DNA/NET plasma concentrations and clinical variables. Differences were assumed to be statistically significant at a level of 5%.

## Results

### Degradation of systemic cf-DNA/MPO (NET) increased the bacterial load during polymicrobial sepsis

**Fig 1:**
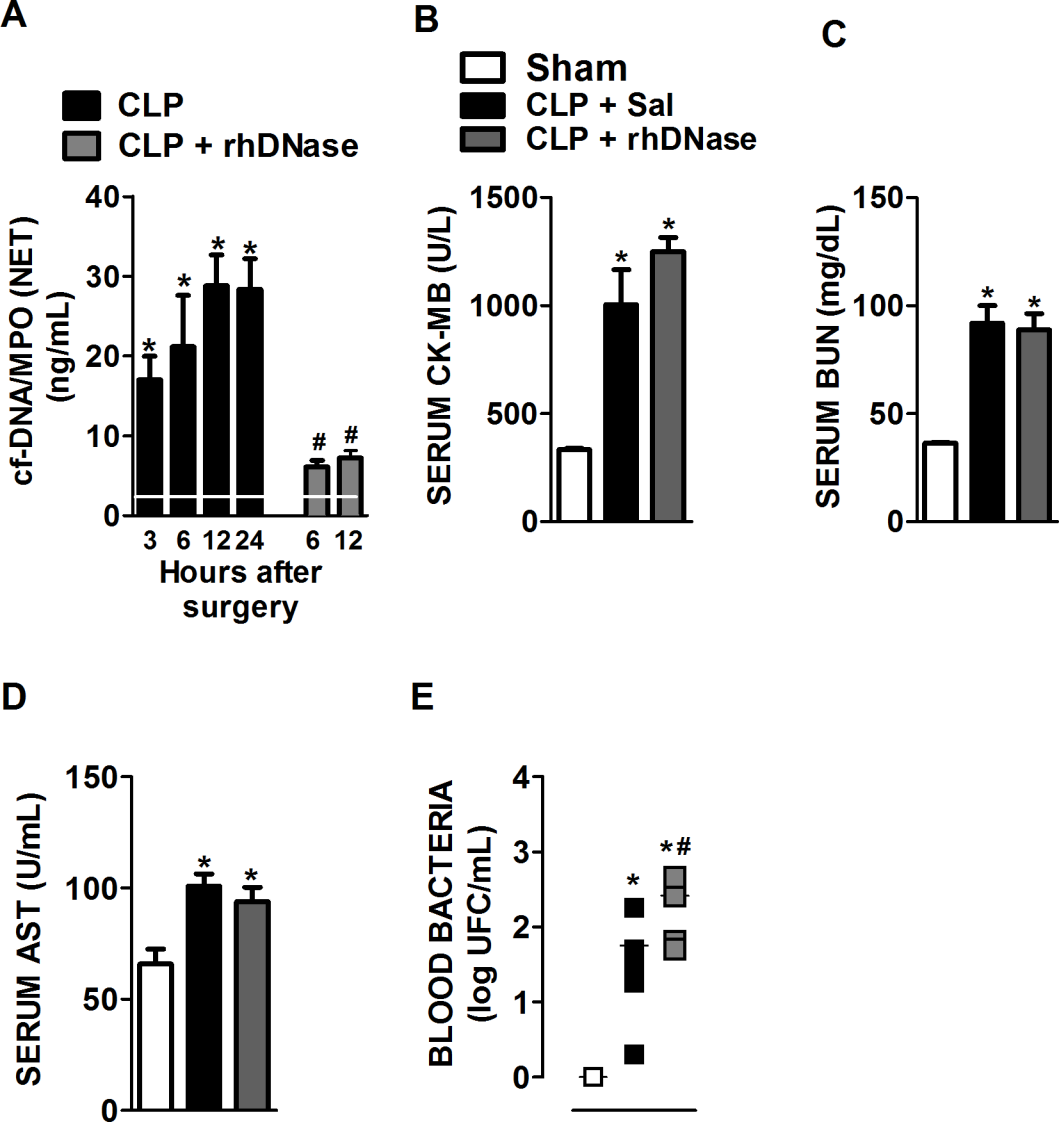
Degradation of systemic cf-DNA/NET by rhDNase treatment did not prevent organ damage during polymicrobial sepsis. Mice were subjected to sham surgery or CLP-induced severe sepsis. (**A**) Blood samples were collected 3, 6, 12 and 24 hours after sepsis induction, and plasma concentrations of cf-DNA/MPO (NET) were determined (white horizontal bar represents the sham group at the indicated times). Blood samples were also collected 6 and 12 hours after sepsis induction in mice treated pre-sepsis (10 min) and post-sepsis (4 h) with rhDNase (10 mg/kg, sc.) * p < 0.05 compared with the sham group, white line; # p < 0.05 compared to the CLP- Sal 6 and 12 h groups (ANOVA followed by Tukey’s test, n = 5 per experimental group). (B) Animals were treated pre-sepsis (10 min) and post-sepsis (4 h) with Sal (control) or rhDNase (10 mg/kg, sc.). Six hours after sepsis induction, the serum levels of CK-MB, BUN (C) and AST (D) were quantified. Blood bacterial levels 6 h (E) were also quantified; # p < 0.05 compared with the CLP-Sal 6 h group (ANOVA followed by Tukey’s test, n = 5 per experimental group). The horizontal bars represent the median (Mann-Whitney U test, n = 5–7 per experimental group). The data are reported as the mean ± SEM. * p < 0.05 compared with the sham group; # p < 0.05 compared with the CLP+Sal group (ANOVA followed by Tukey’s test, n = 5 per experimental group).

We investigated the systemic formation of NETs in mice following CLP-induced sepsis. As shown in Fig 1A, the plasma concentrations of cf-DNA/MPO (NET) were higher in septic mice than in sham mice. A significant increase in the plasma concentration of NETs was observed 3 h after CLP-surgery, increasing thereafter until 24 h (last measurement). The NETs levels were similar to the cf-DNA concentrations measured in the plasma (S1A Fig). Additionally, the serum and plasma concentrations of cf-DNA and NETs were similar (S1B and S1C Fig). Moreover, sepsis-induced high bacteremia (S2A Fig) and an intense systemic inflammatory response characterized by elevated serum concentrations of TNF-α (S2B Fig), CK-MB (S2D Fig), BUN (S2E Fig) and AST (S2F Fig) and increased neutrophil accumulation in the lungs, indicated by increased MPO activity (S2C Fig). Moreover, septic mice showed 100% mortality by day 2, while all sham group mice survived until day 7 (S2G Fig). Together, these results indicate that severe sepsis induced systemic NETs formation and the development of organ dysfunction.

**Fig 2:**
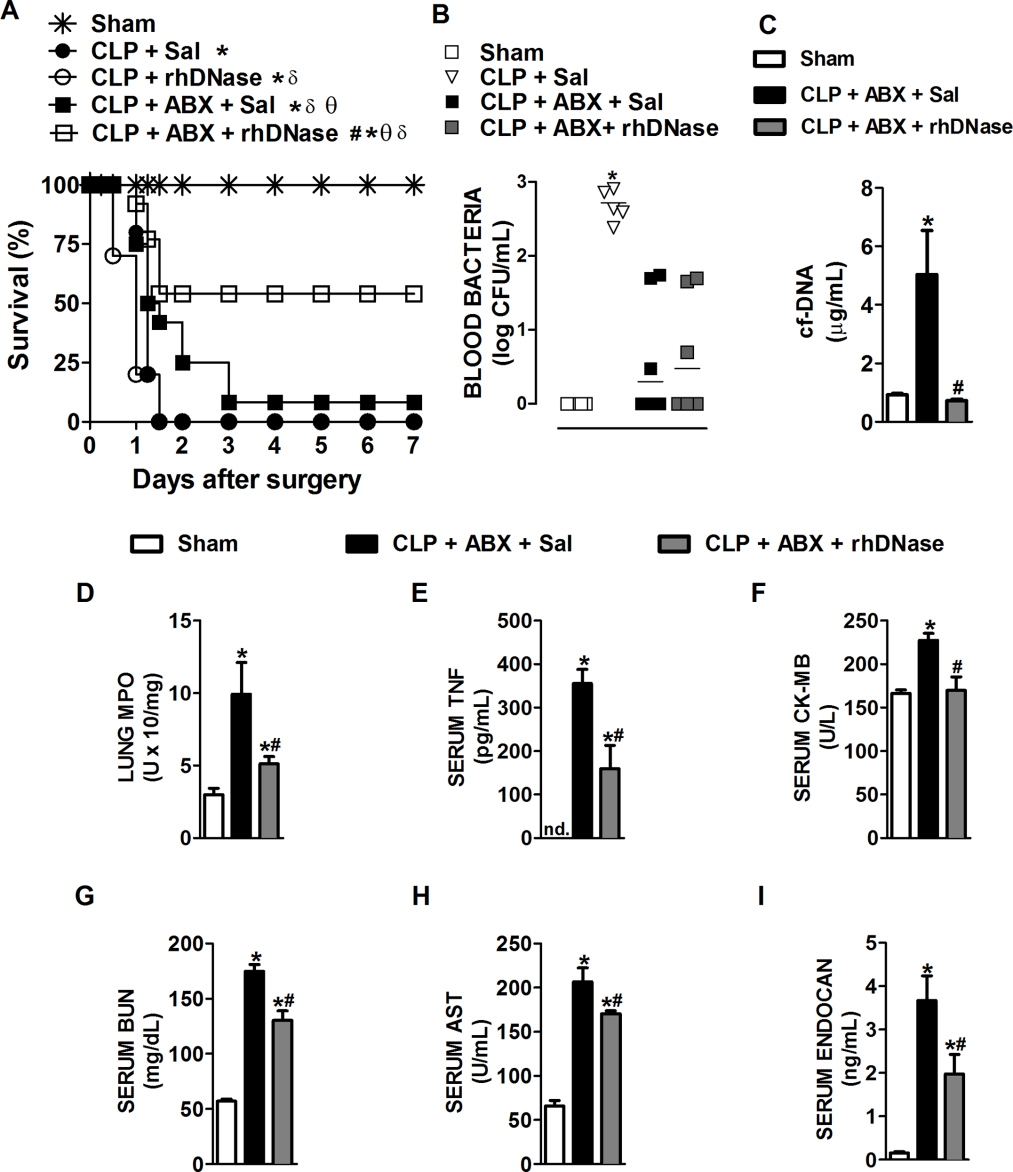
NETs degradation by rhDNase treatment associated with antibiotic therapy improves the outcome of CLP-induced sepsis. Mice were subjected to sham surgery or CLP-induced severe sepsis. (**A**) Mice were post-treated with saline or rhDNase (10 mg/kg, sc. - 1 h after the surgery and every 8 h thereafter), associated or not with ertapenem antibiotic (ABX—30 mg/kg, sc. - 1 h after the surgery and every 12 h thereafter for 48 h). The survival rates were evaluated over 7 days. * p < 0.05 compared with the sham group; δ p < 0.05 compared with the CLP + Sal group; θ p < 0.05 compared with the CLP + rhDNase group; # p < 0.05 compared with the CLP + ABX + Sal group (Mantel-Cox log-rank test, n = 10 per experimental group). (**B-I**) Mice were post-treated with saline or rhDNase (10 mg/kg, sc. - 1 and 8 h after the surgery), associated or not with ertapenem antibiotic (30 mg/kg, sc. - 1 h after the surgery). Twelve hours after sepsis induction, blood bacterial levels (**B**), serum concentration of cf-DNA, (**C**), MPO in lung tissue (**D**) and serum concentrations of TNF **(E**), CK-MB (**F**), BUN (**G**), AST (**H**) and endocan (**I**) were quantified. The data are reported as the mean ± SEM. * p < 0.05 compared with the sham group; # p < 0.05 compared with the CLP + ABX + Sal group (ANOVA followed by Tukey’s test, n = 5 per experimental group); bacteria: the horizontal bars represent the median (Mann-Whitney U test, n = 5–7 per experimental group).

To verify whether NETs were involved in the development of organ dysfunction and control of bacterial growth in our experimental conditions, we investigated whether the degradation of DNA, the major constituent of NETs [10], affected the sepsis-induced organ lesions and the bacterial load. For this purpose, mice were pre-treated with rhDNase (10 mg/kg, sc.) 10 min before CLP-induced sepsis and post-treated every 8 hours. This dose was chosen after a dose-response study (data not shown). The treatment of septic mice with rhDNase significantly decreased their plasma concentrations of cf-DNA/MPO (NET) compared to those of the untreated septic mice (Fig 1A) and of cf-DNA (S1A Fig). We did not observe an attenuation of cardiac, renal or hepatic lesions in septic mice treated with rhDNase, which had serum concentrations of CK-MB (Fig 1B), BUN (Fig 1C) and AST (Fig 1D) similar to septic mice treated with saline. Moreover, the degradation of NETs increased the bacterial load in the blood of septic mice compared to septic mice treated with saline (Fig 1E). Accordingly, septic mice treated with rhDNase started to die earlier than septic mice treated with saline (Fig 2A). The treatment with the dose of 10 mg/kg (iv.) or 5 mg/kg (either iv. or sc.) of rhDNase also reduced the serum concentrations of cf-DNA (data not shown).

### Treatment with rhDNase plus antibiotic improves CLP-induced severe sepsis outcomes

Next, we asked whether post-treatment of septic mice with rhDNase combined with antibiotics to control the infection would allow us to determine a possible role for NETs in sepsis-induced organ injury. Thus, mice subjected to severe sepsis were treated with ertapenem (30 mg/kg, sc. - 1 h after the surgery and every 12 h thereafter) alone or with ertapenem plus rhDNase (10 mg/kg, sc. - 1 h after the surgery and every 8 h for 48 h). We observed that the septic mice treated with antibiotics had reduced blood bacterial counts compared to septic mice treated with saline (Fig 2B). Combined treatment (antibiotics plus rhDNase) led to a reduction in cf-DNA (Fig 2C), together with control of the bacterial burden (Fig 2B). Additionally, as observed in Fig 1C, rhDNase plus ertapenem significantly increased the survival rate when compared to the septic group treated with ertapenem alone. The overall improvement in survival was associated with a reduced systemic inflammatory response and organ injury. Mice treated with saline plus antibiotics had higher MPO activity in the lungs (Fig 2D) and higher serum levels of TNF (Fig 2E), CK-MB (Fig 2F), BUN (Fig 2G) and AST (Fig 2H) compared with the sham group. We also observed high serum concentrations of endocan (Fig 2I), a proteoglycan expressed by endothelial cells of lung and kidney tissues that are released into the blood when endothelial damage occurs [36]. Septic mice treated with rhDNase plus ertapenem showed a reduction in all parameters of injury analyzed (Fig 2D–2I), suggesting that NETs mediate systemic tissue damage during severe sepsis. We also evaluated the inflammatory response in the peritoneal cavity, which was considered the primary infection focus. We observed that septic mice treated with ertapenem plus rhDNase had similar amounts of mononuclear leukocytes (S3A Fig) and neutrophils (S3B Fig) compared to septic mice treated with ertapenem alone.

### Systemic cf-DNA degradation attenuated organ damage during LPS-induced endotoxic shock

**Fig 3:**
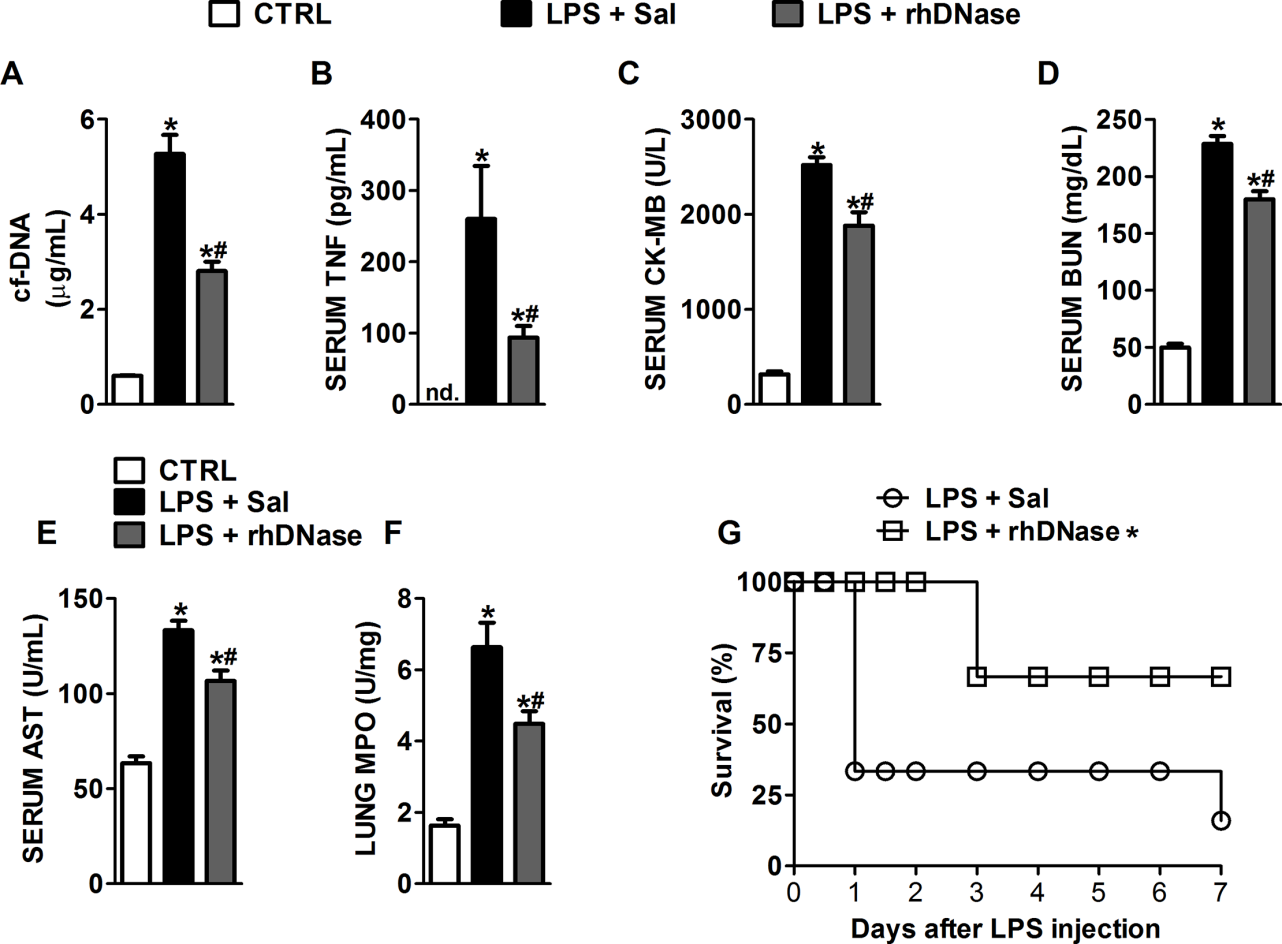
Systemic degradation of NETs attenuated organ damage during LPS-induced endotoxic shock. Endotoxic shock was induced by LPS injection (15 mg/kg, iv.) Mice were pre-treated (10 min) and post-treated (8 h) with saline or rhDNase (10 mg/kg, sc.). Twelve hours after endotoxic shock induction, the serum concentrations of cf-DNA (**A**) and TNF-α (**B**), serum levels of serum CK-MB (**C**), BUN (**D**), AST (**E**) and MPO in lung tissue (**F**) were determined. The data are reported as the mean ± SEM. * p < 0.05 compared with the sham group; # p < 0.05 compared with the LPS+Sal group (ANOVA followed by Tukey’s test, n = 5 per experimental group). (**G**) Survival rates of endotoxemic mice pre-treated (10 min) and post-treated (8/8 h) with saline or rhDNase (10 mg/kg, sc.) until the 48^th^ hour. * p < 0.05 compared with the LPS+Sal group (Mantel-Cox log-rank test, n = 10 per experimental group).

To confirm our results, we investigated whether the rhDNase treatment could decrease organ dysfunction in LPS-induced endotoxic shock, a model without live bacteria [37]. For that, endotoxic shock was induced by LPS administration (15 mg/kg, iv.), and the mice were treated with saline or rhDNase (10 mg/kg, sc.) 10 min and 8 hours after LPS administration. In this case, we used pre-treatment because LPS has a very fast absorption rate and produces a systemic inflammatory response a few minutes after its administration. Twelve hours after endotoxic shock induction, the mice had high serum concentrations of cf-DNA/NET (Fig 3A), TNF (Fig 3B), CK-MB (Fig 3C), BUN (Fig 3D), and AST (Fig 3E), as well as high MPO activity in lung tissue (Fig 3F), compared to the control mice. The rhDNase treatment reduced all these parameters. Moreover, treatment of the endotoxemic mice with rhDNase 10 min after LPS administration and then every 8 hours thereafter until day 2 increased the survival (Fig 3G) compared to the saline-treated group.

### Systemic NETs degradation attenuated histopathological injury in the heart, lungs, and liver

**Fig 4:**
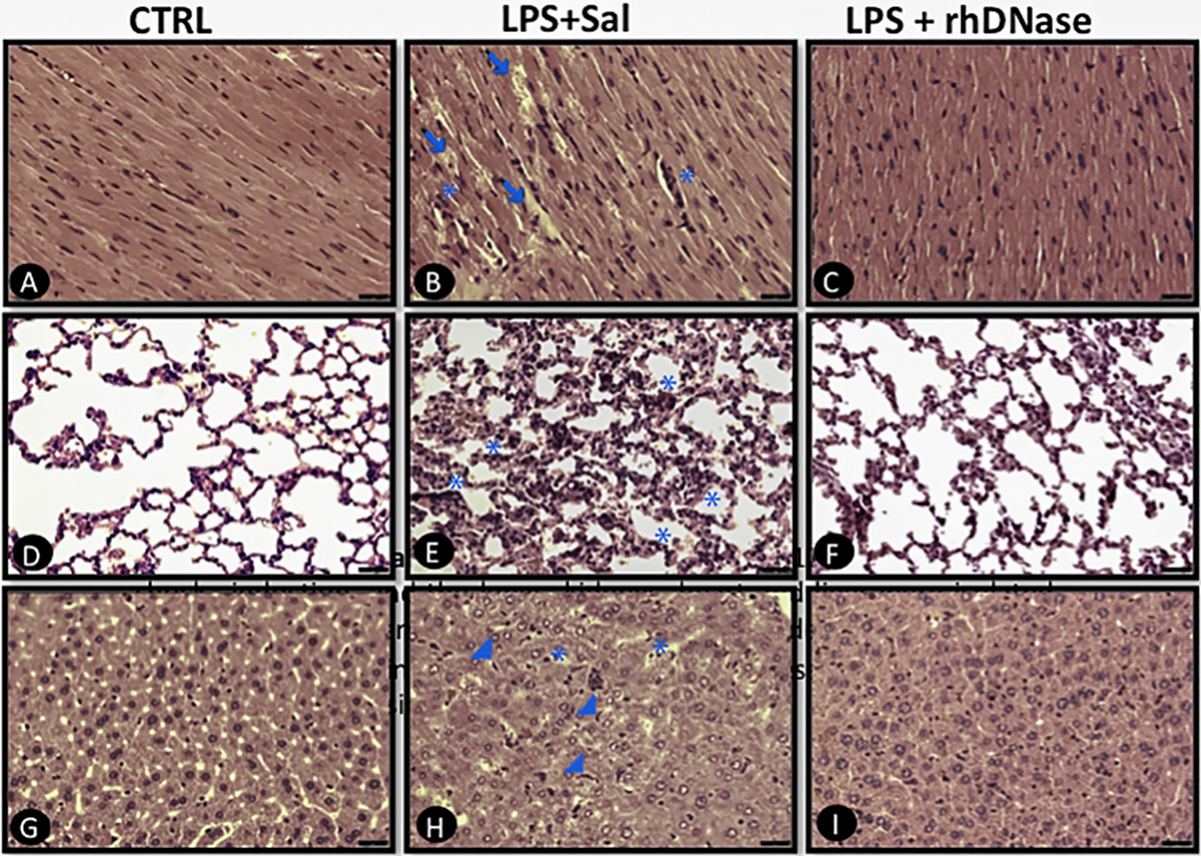
Morphological changes in heart, lungs and liver tissues. Animals were euthanized 12 h after endotoxic shock induction, and the heart, lungs, and liver were isolated, fixed by immersion in 10% paraformaldehyde, dehydrated and embedded in paraffin wax. Then, 5-μm-thick sections were stained with hematoxylin and eosin for histological examination. The images are representative of heart, lung and liver sections from the CTRL (**A, D, G**), LPS+Sal (**B, E, H**) and LPS+rhDNase (**C, F, I**) groups. Arrows indicate edema. Stars indicate leukocyte infiltration. Arrowheads indicate hyperplasia and hypertrophy of Kupffer cells. n = 5 per each experimental group.

We next performed histological analyses to confirm the biochemical markers of lesion findings. Normal (score 0) heart (Fig 4A), lungs (Fig 4D) and liver (Fig 4G) were detected in the control group. In contrast, hearts from the LPS+Sal mice were significantly damaged, with extended myocardial bundles, edematous dissociation (+++), congestion of the capillary beds (++) and the presence of mononuclear and polymorphonuclear leukocytes attached to the vascular endothelium (++). Moreover, the endotoxemic mice presented wide cardiomyocytes, enlarged intercalated discs and dissociated myofibrils and nuclear chromatin (Fig 4B). These histological changes were less pronounced in the LPS+rhDNase group, which showed interstitial edema (++), normal cardiomyocytes and few leukocytes in the tissue (++) (Fig 4C). The LPS+Sal group exhibited pronounced histological changes in the lungs that included thickening of the septum (++), edema (++), congestion (+++) and high leukocyte infiltration into the interstitium (++) (Fig 4E). The rhDNase treatment attenuated these histological changes, with less severe thickening of the septum (+), edema (+), congestion (+) and interstitial leukocyte infiltration (+) (Fig 4F). The LPS+Sal group also exhibited pronounced histological changes in the liver, characterized by enlarged sinusoids (+++), increased volume of endothelial cells with rounded nuclei (++), and a high number of leukocytes in the lumen (++). Moreover, we observed Kupffer cell hypertrophy and hyperplasia (+++) along with the presence of leukocytes (++) close to periportal areas and congestion of the central vein with swollen hepatocytes (Fig 4H). These histological changes were inhibited in the LPS+rhDNase group, which presented characteristics similar to those of the control group (Fig 4I).

**Fig 5:**
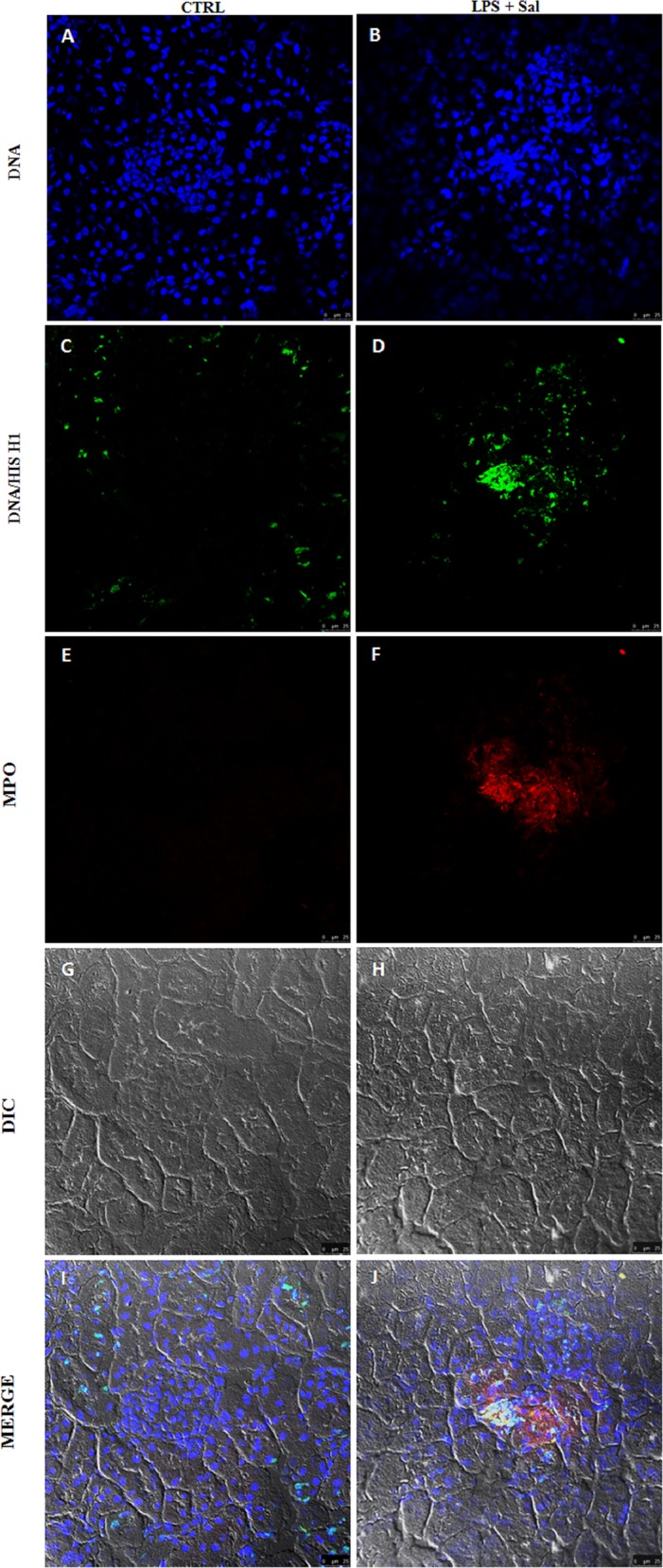
LPS-induced endotoxic shock leads to NETs deposition in kidney tissue. Endotoxic shock was induced by LPS injection (15 mg/kg, iv.). In situ immunofluorescence analysis of frozen tissue sections of kidney harvested 12 h after endotoxic shock induction was conducted. (**A-B**) DNA stained. (**C-D**) DNA/histones stained. (**E-F**) MPO stained. (**G-H**) Tissue structure was visualized by differential interference contrast (DIC) microscopy. NETs were identified by the co-localization of DNA, histone and MPO markers. (**I-J**) Co-localization of DNA (blue), DNA/histone (green) and MPO (red) indicates intraglomerular NETs formation. n = 5 per experimental group.

Further, we evaluated whether NETs are present in sepsis-related injured tissues. For that, we performed immunofluorescence and confocal microscopy analysis of kidney tissues 12 h after the induction of endotoxic shock. We found the typical components of NETs, i.e., DNA/histones (Fig 5D) and MPO (Fig 5F), in the kidneys of endotoxemic mice but not in the kidneys of control mice (Fig 5A, 5C and 5E).

### Cf-DNA/NET correlates with organ failure in septic patients

To assess whether our experimental observations could be extended to the clinical setting, we quantified the serum concentrations of cf-DNA from healthy control volunteers and septic patients. We prospectively included 31 patients within the first twenty-four hours of admission in the Emergency Department of a high-complexity hospital. Eight healthy volunteers were included as controls. The baseline demographic and clinical characteristics are summarized in S1 Table. The majority of the patients were classified as having septic shock (74.19%). Additionally, we observed high SOFA (mean = 12, SD = 5.75) and APACHE II (mean = 25.5, SD = 9.75) scores, as well as low survival rates (in-hospital mortality of 61.29%).

**Fig 6:**
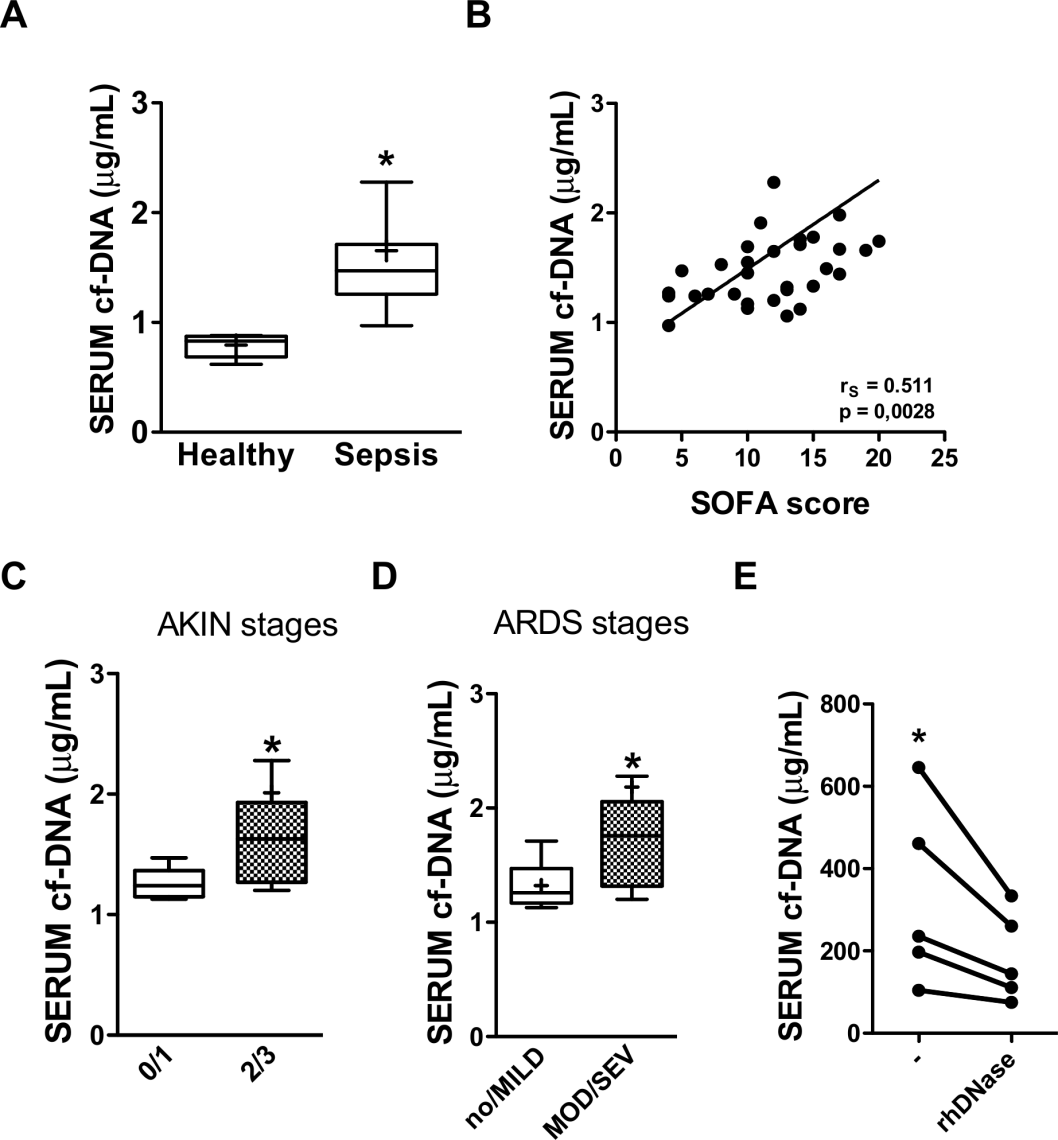
Serum concentrations of cf-DNA are positively correlated with organ failure in septic patients. (**A**) Cf-DNA was quantified in serum samples obtained from healthy control volunteers (n = 8) and septic patients (n = 31). * p < 0.05 compared with healthy control volunteers (Mann-Whitney U test). (**B**) Correlation of cf-DNA serum concentrations with SOFA scores. Serum cf-DNA concentration according to stages of (**C**) AKIN score and (**D**) ARDS, according to the Berlin Definition. *p < 0.05 compared with 0/1 stages (Mann-Whitney U test). (**E**) Patient serum samples incubated with rhDNase for 30 minutes. *p < 0.05 compared with serum+rhDNase (Paired t-test). AKIN—Acute Kidney Injury Network; ARDS—Acute Respiratory Distress Syndrome; no—no ARDS; rS–Spearman’s rho; SOFA–Sequential Organ Failure Assessment.

Consistent with the results obtained in murine experiments, septic patients showed higher serum concentrations of cf-DNA than healthy control volunteers (Fig 6A). We did not observe differences in the serum concentrations of cf-DNA from severe septic and septic shock patients (data not shown). Therefore, we examined the correlation of cf-DNA with organ dysfunction as assessed by the SOFA score. We found a significant positive correlation between the two parameters (rS = 0.511, p = 0.0028) (Fig 6B), demonstrating that cf-DNA increased together with the severity of organ dysfunction. Then, we found that cf-DNA was higher in patients who developed AKIN 2 or 3 acute kidney injury (Fig 6C). In addition, lung injury correlated with cf-DNA, as higher concentrations of cf-DNA were found in patients who developed moderate or severe ARDS compared to patients without ARDS or with mild ARDS (Fig 6D). Additionally, we found a positive correlation between increasing serum concentrations of cf-DNA and bilirubin plasma concentrations (rS = 0.43; p = 0.03—data not shown). Furthermore, to confirm that we measured cf-DNA in our samples, we investigated whether rhDNase could reduce the concentration of cf-DNA in the serum of the septic patients. Indeed, as depicted in Fig 6E, cf-DNA concentrations were reduced following a single in vitro incubation with rhDNase.

## Discussion

In this study, we demonstrated that cf-DNA/NETs are present in septic patients and in mice with CLP-induced experimental sepsis. We noticed that when cf-DNA, a major constituent of NETs structure, was degraded in CLP-induced sepsis by rhDNase treatment, the animals began to die earlier than in the control group, possibly due to a blockage of bacterial burden control in those animals. This observation is in agreement with a previous study reporting that NETs depletion precludes the early immune response and aggravates polymicrobial sepsis [38]. Moreover, Luo and colleagues have shown that NETs exert a proinflammatory role in sepsis, which is associated with organ injury [39]. This scenario creates an apparent paradox in which NETs are important for bacterial clearance as they simultaneously create an environment of tissue damage.

To circumvent this issue, we decided to combine rhDNase treatment with a broad-spectrum antibiotic (ertapenem) to avoid a failure of bacterial control. This strategy prevented the rhDNase-related increase in the bacterial burden, decreased sepsis-induced organ damage, and increased the overall survival rate of septic mice. All assessed parameters of organ injury (CK-MB, BUN, AST), endothelial damage (endocan), systemic inflammation (serum TNF-α and MPO in the lungs) and histopathological changes were improved by the combined treatment, demonstrating that the effects are not organ specific. Additionally, we evaluated the inflammatory response at the focus of infection and did not observe any difference in the number of mononuclear cells or neutrophils. These results indicate that the improved outcome of septic mice treated with antibiotics plus rhDNase was not due to better control of the local infection but instead due to a systemic effect of the treatment. Accordingly, the decrease in NETs following rhDNase treatment also reduced the levels and intensity of inflammatory and organ injury parameters in the aseptic model of LPS-induced endotoxic shock. Similarly, other studies also demonstrated that rhDNase treatment reduced organ injury in CLP- or LPS-induced sepsis [40, 41]. However, different to our study, these authors did not observe a relevant role for NETs in the control of bacterial spreading [40, 27].

To measure cf-DNA, a major constituent of NETs and cf-DNA associated with MPO (cf-DNA/MPO), another constituent of NETs, we used two methodologies and demonstrated that, in addition to the levels of free DNA (cf-DNA) being higher in relation to the levels of cf-DNA/MPO in the serum, both methods show similar results for the release after sepsis induction. Thus, our data indicate that the use of the cf-DNA method can be a surrogate to indicate NETs formation. In contrast to our results, Hamaguchi and colleagues have previously demonstrated that NETs may not be the primary source of cf-DNA and hypothesize that necrotic tissues are responsible for cf-DNA release [42]. However, whether neutrophil-derived traps are the main source of cf-DNA and in which exact situations this might occur are still a matter of debate.

NETs are highly related to endothelial damage and organ failure, which are important events in sepsis. Activated endothelial cells induce the formation of NETs *in vitro*, promoting damage to themselves [41, 43]. Additionally, Clark and colleagues showed that neutrophil interaction with activated platelets during sepsis also causes NETs formation, which contributes to endothelial cell damage and organ injuries [44]. Notwithstanding, circulating cell-free NETs can adhere to the vascular endothelium in both venules and arterioles in LPS-induced endotoxic shock [25]. Although the mechanism is unknown, it was reported that histones and myeloperoxidase, critical components of NETs, could be responsible for NETs-induced endothelial dysfunction [42, 45, 46]. Moreover, it was demonstrated that histones can interact directly with TLR2 and TLR4 to induce cell signaling via MyD88, NF-κB and MAPK and cytokine production. Therefore, we demonstrated that after LPS-induced endotoxic shock there is deposition of NETs in the kidneys of mice, which could have contributed to tissue injury, as previously demonstrated [46, 47]. Our results suggest that better outcomes for severe sepsis and endotoxic shock might also occur due to the decreased availability of damage-associated molecular pattern molecules (DAMPs) that are obtained by NETs degradation, probably reducing the activation of immune cells via TLRs. Consequently, the rhDNase-treated septic mice showed a lessened systemic inflammatory response, therefore resulting in fewer tissue lesions compared with the saline-treated septic mice. Along these lines, it was demonstrated that NETs induced the release of CXCL2, TNF-α and HMGB1 by macrophages [41]. However, other data did not exclude that, at least in part, the beneficial effect of rhDNase treatment could result as a consequence of the prevention of systemic cf-DNA released by other cell types, which acts as a DAMP.

Hashiba and collaborators demonstrated that NETs generation by neutrophils from septic patients is downregulated compared to neutrophils from nonseptic patients [26], which could suggest a possible impairment in NETs formation in septic patients. The authors did not investigate *in vivo* NETs formation in septic patients. In this context, to the best of our knowledge, we are the first to describe that serum NETs are positively correlated to clinical parameters of sepsis severity and organ injury. We demonstrated that septic patients who presented moderate or severe acute kidney injury (AKIN grades 2 and 3, respectively) have increased circulating serum NETs compared to those without acute kidney injury or with only mild disease (AKIN grade 1). Additionally, NETs levels were higher in subjects that developed severe or moderate ARDS compared to those with mild ARDS or without ARDS. Accordingly, NETs were previously related to ARDS development in patients with influenza pneumonitis [19].

Some potential limitations of this work include the use of an experimental model with 100% mortality, which does not perfectly model the general clinical setting. However, it is important to note that even in this severe experimental condition, the blockage of NETs presented a marked protective effect. One can also argue that another limitation was the use of cf-DNA as a surrogate to NETs in a number of different assays, although we have demonstrated that they strongly correlate with each other. Because we included septic patients from a high-complexity tertiary and reference hospital, a selection bias might have occurred, which resulted in an observed high mortality rate. The control group was healthy volunteers instead of non-septic sick patients. For this reason, based on our data, NETs cannot be used as a biomarker to discriminate septic from non-septic patients. However, as mentioned previously, our data support the hypothesis that NETs levels separate patients with more severe organ-specific damage from those with less severe disease. Moreover, the study power was not strong enough to test the hypothesis that NETs is correlated with mortality in septic patients.

We showed that NETs are present in sepsis and participate in sepsis-induced organ failure. These results encourage us to support the evaluation of serum (or plasma) NETs concentration as a predictive biomarker for the early assessment of sepsis severity. Moreover, our experimental findings lead us to envision a phase I/II clinical trial to test whether degrading cf-DNA/NET through rhDNase administration could improve sepsis outcomes in patients.

## Conclusions

Our data indicate that cf-DNA/NETs are involved in the severity of sepsis in mice and humans. Although NETs play a beneficial role in the killing of pathogens, their involvement in sepsis-induced organ injury is deleterious. Therefore, the strategy of rhDNase degradation of cf-DNA coupled with antibiotic therapy might represent a novel therapy for the management of sepsis and the prevention of organ dysfunction.

## Supporting Information

S1 Fig Cf-DNA correlates with NET concentration in plasma and serum. Mice were subjected to sham or CLP-induced severe sepsis. At the indicated time-points, cf-DNA (A-B) or NET (C) were measured in plasma or serum, as indicated in the figure. (A) Blood samples were collected 3, 6, 12 and 24 hours after sepsis induction, and plasma concentrations of cf-DNA were determined (horizontal white bar represents the sham group at the indicated times). * p < 0.05 compared with the sham group (ANOVA followed by Tukey’s test, n = 5 per experimental group). Animals were treated pre-sepsis (10 min) and post-sepsis (4 h) with Sal (control) or rhDNase (10 mg/kg, *sc*.). Plasma concentrations of cf-DNA 6 and 12 h after sepsis induction and rhDNase treatment (last two bars). (B) Blood samples were collected 6 hours after sepsis induction and cf-DNA levels were determined in plasma and serum. * p < 0.05 compared with the sham group (ANOVA followed by Tukey’s test, n = 5 per experimental group). (C) Blood samples were collected 6 hours after sepsis induction, and NETs were determined in plasma and serum. * p < 0.05 compared with the sham group (ANOVA followed by Tukey’s test, n = 5 per experimental group).
(TIF)

S2 Fig Mice presented systemic multiple-organ lesions during CLP-induced severe sepsis. Mice were subjected to sham surgery or CLP-induced severe sepsis. At the indicated time-points, blood bacterial levels (A), serum TNF (B), MPO in lung tissue (C) and serum concentrations of CK-MB (D), BUN (E) and AST (F) were determined. The data are reported as the mean ± SEM. *p < 0.05 compared with the sham group; # p <0.05 compared with the 3 h group (ANOVA followed by Tukey’s test, n = 10 per experimental group). Bacteria: * p < 0.05 compared the sham group; # p < 0.05 compared with the 3 h group (Mann-Whitney U test, n = 5 per experimental group). (G) Survival rates of mice subjected to CLP. * p < 0.05 compared with the sham group (Mantel-Cox log-rank test, n = 10 per experimental group).
(TIFF)

S3 Fig Systemic cf-DNA/NET degradation by rhDNase treatment with ertapenem did not alter leukocyte migration into the peritoneal cavity. Mice were subjected to sham surgery or CLP-induced severe sepsis. The mice were post-treated with saline or rhDNase (10 mg/kg, sc. - 1 h after the surgery and every 8 h thereafter) with ertapenem antibiotic (ABX—30 mg/kg, sc. - 1 h after the surgery and every 12 h thereafter). Twelve hours following sepsis induction, the numbers of mononuclear cells (A) and neutrophils (B) were determined in the peritoneal lavage. * p <0.05 compared with the sham group (ANOVA followed by Tukey’s test, n = 5 per experimental group).
(TIFF)

S1 Table Baseline demographic and clinical characteristics of the septic patients. ^§^ NETs (neutrophil extracellular traps) serum concentrations are expressed as μg/mL. Bilirubin and creatinine serum concentrations are expressed as mg/mL.
(DOCX)

## References

[1] Bone RC, Balk RA, Cerra FB, Dellinger RP, Fein AM, Knaus WA et al Definitions for sepsis and organ failure and guidelines for the use of innovative therapies in sepsis. The ACCP/SCCM Consensus Conference Committee. American College of Chest Physicians/Society of Critical Care Medicine. Chest. 1992;101: 1644–55. 1303622 10.1378/chest.101.6.1644

[2] Remick DG. Pathophysiology of sepsis. Am J Pathol. 2007; 170: 1435–44. 17456750 10.2353/ajpath.2007.060872 PMC1854939

[3] Bachelerie F, Ben-Baruch A, Burkhardt AM, Combadiere C, Farber JM, Graham GJ, et al International Union of Pharmacology. LXXXIX. Update on the Extended Family of Chemokine Receptors and Introducing a New Nomenclature for Atypical Chemokine Receptors. Pharmacol Rev. 2014; 66: 1–79. 10.1124/pr.113.007724 24218476 PMC3880466

[4] Canetti C, Silva JS, Ferreira SH, Cunha FQ. Tumour necrosis factor-alpha and leukotriene B4 mediate the neutrophil migration in immune inflammation. Br J Pharmacol. 2001;134: 1619–28. 11739237 10.1038/sj.bjp.0704403 PMC1572894

[5] Kaplan MJ, Radic M. Neutrophil extracellular traps: double-edged swords of innate immunity. J Immunol. 2012;189: 2689–95. 10.4049/jimmunol.1201719 22956760 PMC3439169

[6] Aird WC. The role of the endothelium in severe sepsis and multiple organ dysfunction syndrome. Blood. 2003;101: 3765–77. 12543869 10.1182/blood-2002-06-1887

[7] Gustot T. Multiple organ failure in sepsis: prognosis and role of systemic inflammatory response. Curr Opin Crit Care. 2011;17: 153–59. 10.1097/MCC.0b013e328344b446 21346564

[8] Hotchkiss RS, Karl IE. The pathophysiology and treatment of sepsis. N Engl J Med. 2003; 348: 138–50. 12519925 10.1056/NEJMra021333

[9] Hoesel LM, Neff TA, Neff SB, Younger JG, Olle EW, Gao H, et al Harmful and protective roles of neutrophils in sepsis. Shock. 2005; 24(1): 40–7. 15988319 10.1097/01.shk.0000170353.80318.d5

[10] Brinkmann V, Reichard U, Goosmann C, Fauler B, Uhlemann Y, Weiss DS, et al Neutrophil extracellular traps kill bacteria. Science. 2004; 303: 1532–35. 15001782 10.1126/science.1092385

[11] Guimarães-Costa AB, Nascimento MT, Froment GS, Soares RP, Morgado FN, Conceição-Silva F, et al Leishmania amazonensis promastigotes induce and are killed by neutrophil extracellular traps. Proc Natl Acad Sci USA. 2009;106: 6748–53. 10.1073/pnas.0900226106 19346483 PMC2672475

[12] Urban CF, Reichard U, Brinkmann V, Zychlinsky A. Neutrophil extracellular traps capture and kill Candida albicans yeast and hyphal forms. Cell Microbiol. 2006;8: 668–76. 16548892 10.1111/j.1462-5822.2005.00659.x

[13] Bianchi M, Hakkim A, Brinkmann V, Siler U, Seger RA, Zychlinsky A, et al Restoration of NET formation by gene therapy in CGD controls aspergillosis. Blood. 2009; 114: 2619–22. 10.1182/blood-2009-05-221606 19541821 PMC2756123

[14] Fuchs TA, Abed U, Goosmann C, Hurwitz R, Schulze I, Wahn V, et al Novel cell death program leads to neutrophil extracellular traps. J Cell Biol. 2007;176: 231–241. 17210947 10.1083/jcb.200606027 PMC2063942

[15] Bianchi M, Niemiec MJ, Siler U, Urban CF, Reichenbach J. Restoration of anti-Aspergillus defense by neutrophil extracellular traps in human chronic granulomatous disease after gene therapy is calprotectin-dependent. J Allergy Clin Immunol. 2011;127(5): 1243–52. 10.1016/j.jaci.2011.01.021 21376380

[16] Young RL, Malcolm KC, Kret JE, Caceres SM, Poch KR, Nichols DP, et al Neutrophil extracellular trap (NET)-mediated killing of Pseudomonas aeruginosa: evidence of acquired resistance within the CF airway, independent of CFTR. PLoS One. 2011;6: e23637 10.1371/journal.pone.0023637 21909403 PMC3164657

[17] Fuchs TA, Brill A, Duerschmied D, Schatzberg D, Monestier M, Myers DD Jr, et al Extracellular DNA traps promote thrombosis. Proc Natl Acad Sci USA. 2010;107: 15880–5. 10.1073/pnas.1005743107 20798043 PMC2936604

[18] Caudrillier A, Kessenbrock K, Gilliss BM, Nguyen JX, Marques MB, Monestier M, et al Platelets induce neutrophil extracellular traps in transfusion-related acute lung injury. J Clin Invest. 2012;122: 2661–71. 10.1172/JCI61303 22684106 PMC3386815

[19] Narasaraju T, Yang E, Samy RP, Ng HH, Poh WP, Liew AA, et al Excessive neutrophils and neutrophil extracellular traps contribute to acute lung injury of influenza pneumonitis. Am J Pathol. 2011;179: 199–210. 10.1016/j.ajpath.2011.03.013 21703402 PMC3123873

[20] Villanueva E, Yalavarthi S, Berthier CC, Hodgin JB, Khandpur R, Lin AM, et al Netting neutrophils induce endotelial damage, infiltrate tissues and expose immunostimulatory molecules in systemic lupus erythematosus. J Immunol. 2011; 187: 538–52. 10.4049/jimmunol.1100450 21613614 PMC3119769

[21] Hakkim A, Fürnrohr BG, Amann K, Laube B, Abed UA, Brinkmann V, et al Impairment of neutrophil extracellular trap degradation is associated with lupus nephritis. Proc Natl Acad Sci USA. 2010;107: 9813–18. 10.1073/pnas.0909927107 20439745 PMC2906830

[22] Kessenbrock K, Krumbholz M, Schönermarck U, Back W, Gross WL, Werb Z, et al Netting neutrophils in autoimmune small-vessel vasculitis. Nat Med. 2009;15: 623–25. 10.1038/nm.1959 19448636 PMC2760083

[23] Wong SL, Demers M, Martinod K, Gallant M, Wang Y, Goldfine AB, et al Diabetes primes neutrophils to undergo NETosis, which impairs wound healing. Nat Med. 2015;21(7):815–9. 10.1038/nm.3887 26076037 PMC4631120

[24] Fattahi F, Grailer JJ, Jajou L, Zetoune FS, Andjelkovic AV, Ward PA. Organ distribution of histones after intravenous infusion of FITC histones or after sepsis. Immunol Res. 2015;61(3):177–86. 10.1007/s12026-015-8628-2 25680340 PMC4339508

[25] Tanaka K, Koike Y, Shimura T, Okigami M, Ide S, Toiyama Y, et al In vivo characterization of neutrophil extracellular traps in various organs of a murine sepsis model. PLoS One. 2014;9(11):e111888 10.1371/journal.pone.0111888 25372699 PMC4221155

[26] Hashiba M, Huq A, Tomino A, Hirakawa A, Hattori T, Miyabe H, et al Neutrophil extracellular traps in patients with sepsis. J Surg Res. 2015;194(1):248–54. 10.1016/j.jss.2014.09.033 25438956

[27] Martinod K, Fuchs TA, Zitomersky NL, Wong SL, Demers M, Gallant M, et al PAD4-deficiency does not affect bacteremia in polymicrobial sepsis and ameliorates endotoxemic shock. Blood. 2015;125(12):1948–56. 10.1182/blood-2014-07-587709 25624317 PMC4366625

[28] Levy MM, Fink MP, Marshall JC, Abraham E, Angus D, Cook D, et al SCCM/ESICM/ACCP/ATS/SIS. 2001 SCCM/ESICM/ ACCP/ATS/SIS international sepsis definitions conference. Crit Care Med. 2003;31: 1250–6. 12682500 10.1097/01.CCM.0000050454.01978.3B

[29] Vincent JL, Moreno R, Takala J, Willatts S, De Mendonça A, Bruining H, et al The SOFA (Sepsis-related Organ Failure Assessment) score to describe organ dysfunction/failure. On behalf of the Working Group on Sepsis-Related Problems of the European Society of Intensive Care Medicine. Intensive Care Med. 1996;22: 707–10. 8844239 10.1007/BF01709751

[30] Ferreira FL, Bota DP, Bross A, Mélot C, Vincent JL. Serial evaluation of the SOFA score to predict outcome in critically ill patients. JAMA. 2001;286: 1754–8. 11594901 10.1001/jama.286.14.1754

[31] Lopes JA, Fernandes P, Jorge S, Gonçalves S, Alvarez A, Costa e Silva Z, et al Acute kidney injury in intensive care unit patients: a comparison between the RIFLE and the Acute Kidney Injury Network classifications. Crit Care. 2008;12: R110 10.1186/cc6997 18755026 PMC2575599

[32] Ranieri VM, Rubenfeld GD, Thompson BT, Ferguson ND, Caldwell E, Fan E, et al (ARDS Definition Task Force). Acute respiratory distress syndrome: the Berlin Definition. JAMA. 2012;307: 2526–33. 10.1001/jama.2012.5669 22797452

[33] Hubbard WJ, Choudhry M, Schwacha MG, Kerby JD, Rue LW 3rd, Bland KI, et al Cecal ligation and puncture. Shock. 2005;24(Suppl 1): 52–7. 16374373 10.1097/01.shk.0000191414.94461.7e

[34] Alves-Filho JC, Freitas A, Souto FO, Spiller F, Paula-Neto H, Silva JS, et al Regulation of chemokine receptor by Toll-like receptor 2 is critical to neutrophil migration and resistance to polymicrobial sepsis. Proc Natl Acad Sci USA. 2009;106: 4018–23. 10.1073/pnas.0900196106 19234125 PMC2656197

[35] Margraf S, Lögters T, Reipen J, Altrichter J, Scholz M, Windolf J. Neutrophil-derived circulating free DNA (cf-DNA/NET): a potential prognostic marker for posttraumatic development of inflammatory second hit and sepsis. Shock. 2008;30: 352–8. 10.1097/SHK.0b013e31816a6bb1 18317404

[36] Lassalle P, Molet S, Janin A, Heyden JV, Tavernier J, Fiers W, et al ESM-1 is a novel human endothelial cell-specific molecule expressed in lung and regulated by cytokines. J Biol Chem. 1996;271: 20458–64. 8702785 10.1074/jbc.271.34.20458

[37] Cunha FQ, Assreuy J, Moss DW, Rees D, Leal LM, Moncada S et al Differential induction of nitric oxide synthase in various organs of the mouse during endotoxaemia: role of TNF-alpha and IL-1-beta. Immunology.1994;81:211–5. 7512527 PMC1422329

[38] Meng W, Paunel-Görgülü A, Flohé S, Hoffmann A, Witte I, MacKenzie C, et al Depletion of neutrophil extracellular traps in vivo results in hypersusceptibility of polymicrobial sepsis in mice. Crit Care. 2012;16: R137 10.1186/cc11442 22835277 PMC3580722

[39] Luo L, Zhang S, Wang Y, Rahman M, Syk I, Zhang E, et al Proinflammatory role of neutrophil extracellular traps in abdominal sepsis. Am J Physiol Lung Cell Mol Physiol. 2014;307: L586–96. 10.1152/ajplung.00365.2013 25085626

[40] Gao X, Hao S, Yan H, Ding W, Li K, Li J. Neutrophil extracellular traps contribute to the intestine damage in endotoxemic rats. J Surg Res. 2015;195(1):211–8. 10.1016/j.jss.2014.12.019 25575734

[41] Gupta AK, Joshi MB, Philippova M, Erne P, Hasler P, Hahn S, et al Activated endothelial cells induce neutrophil extracellular traps and are susceptible to NETosis-mediated cell death. FEBS Lett. 2010;584: 3193–7. 10.1016/j.febslet.2010.06.006 20541553

[42] Hamaguchi S, Akeda Y, Yamamoto N, Seki M, Yamamoto K, Oishi K, Tomono K. Origin of circulating free DNA in sepsis: analysis of the CLP mouse model. Mediators Inflamm. 2015;2015:614518 10.1155/2015/614518 26273139 PMC4529942

[43] Saffarzadeh M, Juenemann C, Queisser MA, Lochnit G, Barreto G, Galuska SP, et al Neutrophil Extracellular Traps Directly Induce Epithelial and Endothelial Cell Death: A Predominant Role of Histones. PLoS One. 2012;7(2):e32366 10.1371/journal.pone.0032366 22389696 PMC3289648

[44] Clark SR, Ma AC, Tavener SA, McDonald B, Goodarzi Z, Kelly MM, et al Platelet TLR4 activates neutrophil extracellular traps to ensnare bacteria in septic blood. Nat Med. 2007;13: 463–9. 17384648 10.1038/nm1565

[45] Xu J, Zhang X, Pelayo R, Monestier M, Ammollo CT, Semeraro F, et al Extracellular histones are major mediators of death in sepsis. Nat Med. 2009;15:1318–21. 10.1038/nm.2053 19855397 PMC2783754

[46] Scheibner KA, Lutz MA, Boodoo S, Fenton MJ, Powell JD, Horton MR. Hyaluronan fragments act as an endogenous danger signal by engaging TLR2. J Immunol. 2006;177: 1272–81. 16818787 10.4049/jimmunol.177.2.1272

[47] Allam R, Scherbaum CR, Darisipudi MN, Mulay SR, Hägele H, Lichtnekert J, et al Histones from dying renal cells aggravate kidney injury via TLR2 and TLR4. J Am Soc Nephrol. 2012; 23: 1375–88. 10.1681/ASN.2011111077 22677551 PMC3402284

