# Supplementary figures and images for: Neutrophil Extracellular Traps Induce Organ Damage during Experimental and Clinical Sepsis
Source: PLoS One. 2016 Feb 5;11(2):e0148142. doi: 10.1371/journal.pone.0148142 (PMC4743982; doi:10.1371/journal.pone.0148142)

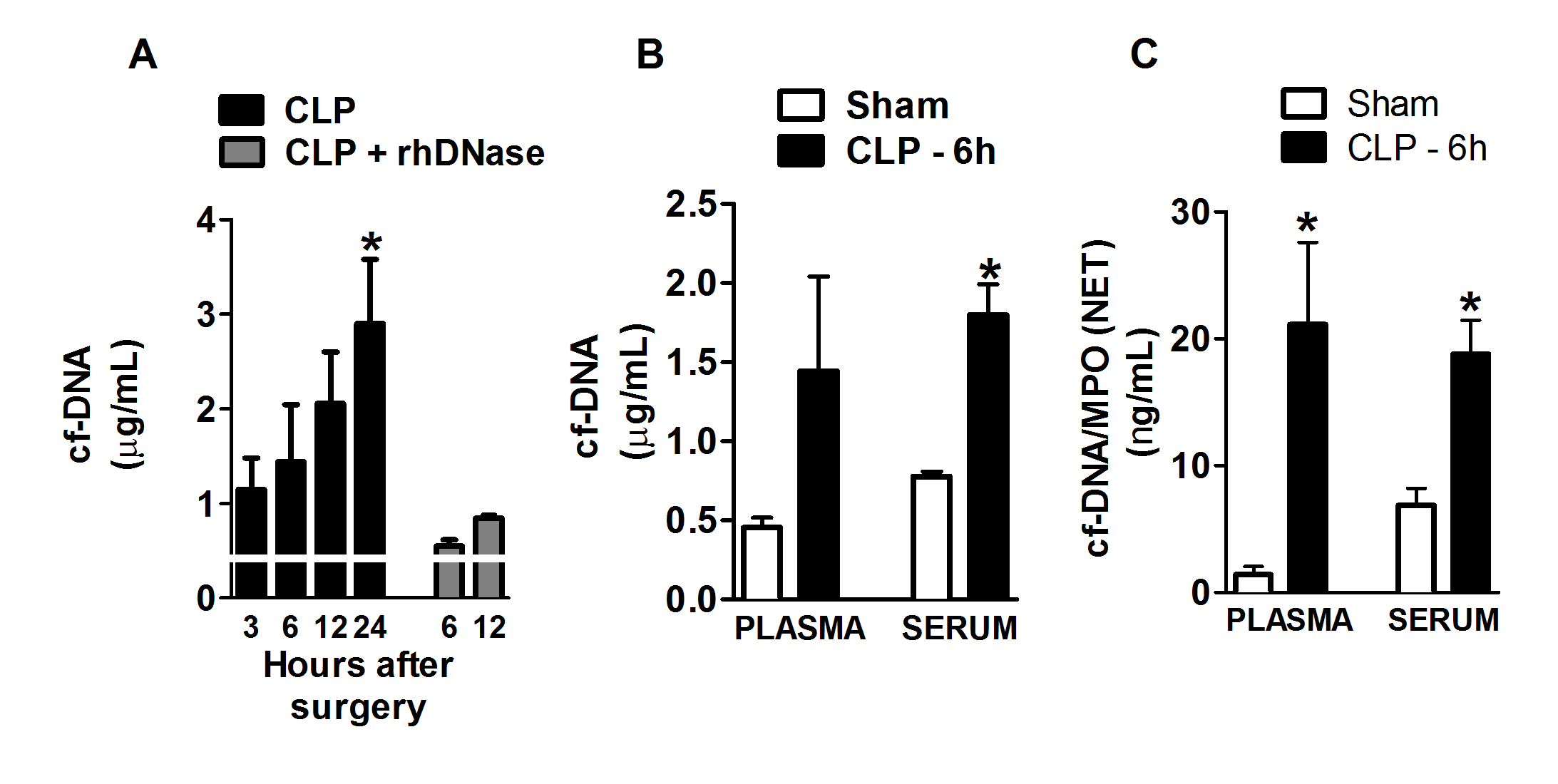

Supplement: S1 Fig — Mice were subjected to sham or CLP-induced severe sepsis. At the indicated time-points, cf-DNA (A-B) or NET (C) were measured in plasma or serum, as indicated in the figure. (A) Blood samples were collected 3, 6, 12 and 24 hours after sepsis induction, and plasma concentrations of cf-DNA were determined (horizontal white bar represents the sham group at the indicated times). * p < 0.05 compared with the sham group (ANOVA followed by Tukey’s test, n = 5 per experimental group). Animals were treated pre-sepsis (10 min) and post-sepsis (4 h) with Sal (control) or rhDNase (10 mg/kg, sc.). Plasma concentrations of cf-DNA 6 and 12 h after sepsis induction and rhDNase treatment (last two bars). (B) Blood samples were collected 6 hours after sepsis induction and cf-DNA levels were determined in plasma and serum. * p < 0.05 compared with the sham group (ANOVA followed by Tukey’s test, n = 5 per experimental group). (C) Blood samples were collected 6 hours after sepsis induction, and NETs were determined in plasma and serum. * p < 0.05 compared with the sham group (ANOVA followed by Tukey’s test, n = 5 per experimental group). (TIF) [file pone.0148142.s001.tif]

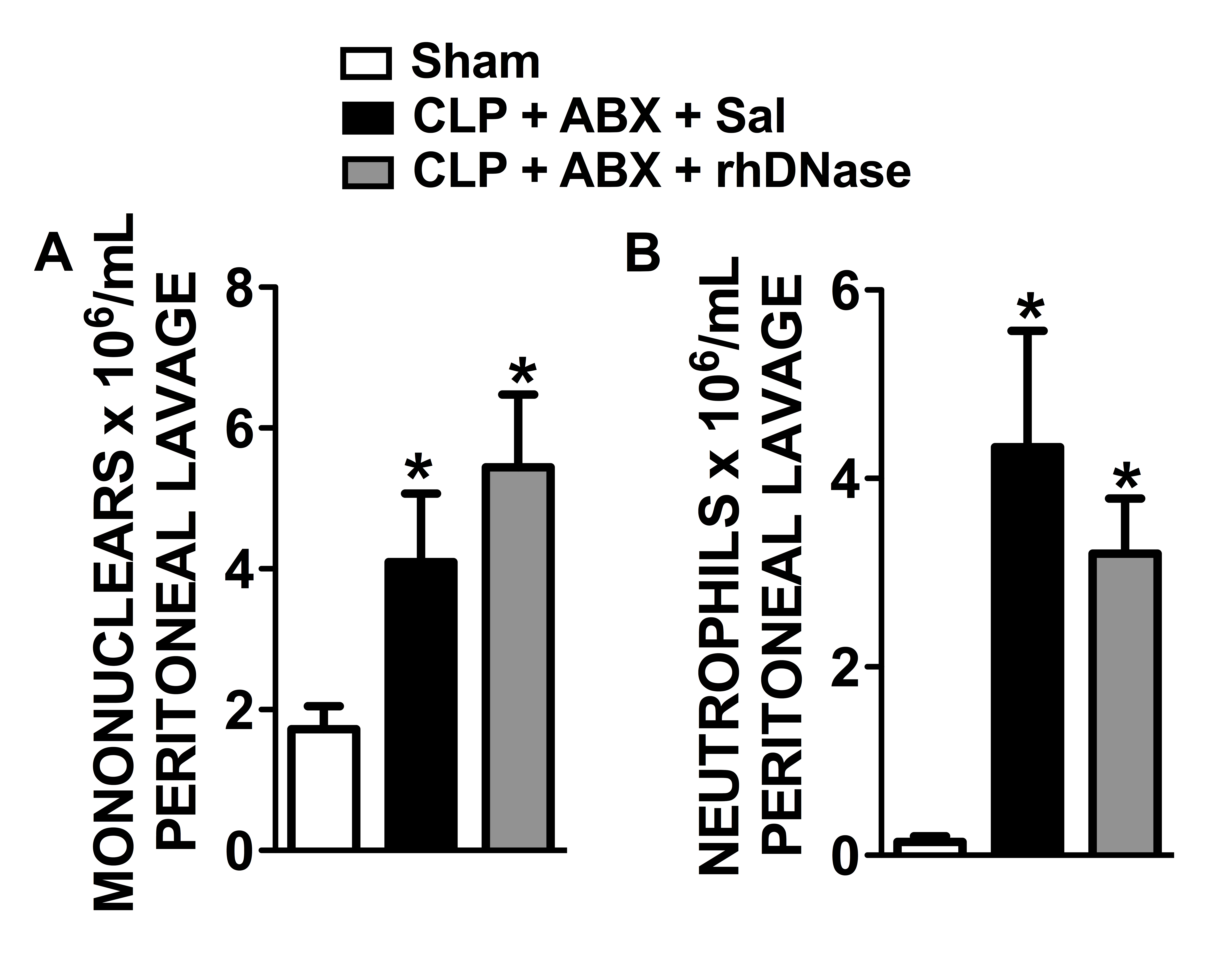

Supplement: S3 Fig — Mice were subjected to sham surgery or CLP-induced severe sepsis. The mice were post-treated with saline or rhDNase (10 mg/kg, sc. - 1 h after the surgery and every 8 h thereafter) with ertapenem antibiotic (ABX—30 mg/kg, sc. - 1 h after the surgery and every 12 h thereafter). Twelve hours following sepsis induction, the numbers of mononuclear cells (A) and neutrophils (B) were determined in the peritoneal lavage. * p <0.05 compared with the sham group (ANOVA followed by Tukey’s test, n = 5 per experimental group). (TIFF) [file pone.0148142.s003.tiff]
