# Supplementary material for: Neutrophil Extracellular Traps Induce Organ Damage during Experimental and Clinical Sepsis
Source: PLoS One. 2016 Feb 5;11(2):e0148142. doi: 10.1371/journal.pone.0148142 (PMC4743982; doi:10.1371/journal.pone.0148142)
Supplement: S1 Table — § NETs (neutrophil extracellular traps) serum concentrations are expressed as μg/mL. Bilirubin and creatinine serum concentrations are expressed as mg/mL. (DOCX) [file pone.0148142.s004.docx]

**Supplementary Table 1** - Baseline demographic and clinical characteristics of the septic patients.

| **Characteristic** |  | **Septic patient (n=31)** |
| --- | --- | --- |
| Mean age - yr |  | 58.54 |
| Female sex - no. (%) |  | 14 (45.1) |
| Severe sepsis - no. (%) |  | 8 (25.81) |
| Septic shock - no. (%) |  | 23 (74.19) |
| APACHE II - mean (SD) |  | 25.5 (9.75) |
| SOFA - mean (SD) |  | 12 (5.75) |
| cf-DNA/NETs - mean (SD) ^§^ |  | 1.64 (0.98) |
| Bilirubin - mean (SD) ^§^ |  | 3.33 (4.7) |
| Creatinine - mean (SD) ^§^ |  | 2.79 (1.7) |
| Site of infection – no. (%) |  |  |
| Lung |  | 14 (45.16) |
| Abdominal |  | 9 (29.01) |
| Other |  | 8 (25.8) |
| Non-survivor - no. (%) |  | 19 (61.29) |

Baseline demographic and clinical characteristics of the septic patients. § means seic concentrations of NETs (neutrophil extracellular traps) are expressed as μg/mL and seric concentrations of bilirubin and creatinine are expressed as mg/mL.
